# Antiobesity Activity of Two Polyherbal Formulations in High-Fat Diet-Induced Obese C57BL/6J Mice

**DOI:** 10.1155/2022/9120259

**Published:** 2022-05-11

**Authors:** Prakash Raj Pandeya, Gopal Lamichhane, Ramakanta Lamichhane, Jiao Luo, Xiao-Jun Li, Su-jin Rhee, Liu Xiangqian, Hyun-Ju Jung

**Affiliations:** ^1^Department of Oriental Pharmacy and Wonkwang-Oriental Medicines Research Institute, Wonkwang University, Sinyong-Dong, Iksan 570-749, Republic of Korea; ^2^School of Pharmacy, Hunan University of Chinese Medicine, Changsha 410208, China; ^3^National Engineering Research Center for Modernization of Traditional Chinese Medicine-Hakka Medical Resources Branch, School of Pharmacy, Gannan Medical University, Ganzhou, Jiangxi 341000, China; ^4^Department of Pharmacy, Wonkwang University, Sinyong-Dong, Iksan 570-749, Republic of Korea

## Abstract

Obesity and overweight have posed a severe threat to humanity, needing urgent efforts for the development of safe and effective therapeutic interventions. In this research work, we have developed two polyherbal formulations A and B basically consisting of *Helianthus tuberosus* root powder (also called inulin of synanthrin) along with other herbs for the treatment of obesity. Evaluation of the antioxidant activity of both formulations using 1,1-diphenyl-2-picrylhydrazyl (DPPH) and 2,2′-azino-bis (3-ethylbenzothiazoline-6-sulfonic acid) (ABTS) free radical scavenging assays showed good antioxidant potentials. Both formulations A and B showed good antiobesity activity on a diet-induced obesity (DIO) model of mice by effectively lowering the body weight of mice compared to the high-fat diet (HFD) control mice, mainly by reducing the food efficiency ratio (FER). Furthermore, both formulations ameliorated lipoprotein misbalances induced by obesity and thus decreased the atherogenic index. Treatment with both formulations significantly decreased the liver and epididymal white adipose tissue (WAT) weight. This was supported by the improvement in steatosis of the liver and reduced hypertrophy in WAT on histological examination. In addition, formulations A and B have been seen as effective in controlling fasting blood glucose levels probably by alleviating HFD-induced insulin resistance. All of these results collectively suggest that formulations A and B serve as potentially safe and effective herbal interventions to control obesity and its comorbidities.

## 1. Introduction

The World Health Organization (WHO) defines obesity as an abnormal or excessive accumulation of fat in the body that may impair the health of people. Adult people having a body mass index (BMI) ≥ 25 are considered overweight while BMI ≥ 30 represents obesity. Obesity was tripled within the last four decades, counting 1.9 billion overweight adults worldwide in 2016, among which 650 million were obese. Child obesity was increased as well, totaling 39 million children under the age of five being overweight or obese in 2020 [1]. The surge in obesity comes together with the burden of healthcare costs associated with it, evidenced by the finding that an obese person expends 30% more on healthcare costs than their normal-weight counterparts [2]. These rising statistics outline the urgency of an appropriate intervention that will control obesity, reducing future healthcare burden related to comorbidities such as hypertension, dyslipidemia, type 2 diabetes, coronary heart disease, stroke, sleep apnea, and some cancers [3].

Obesity treatment is done by three approaches: behavioral and lifestyle modification, pharmacotherapy, and surgery. Behavioral and lifestyle change only works for a preventive approach, but it is difficult to make a significant impact in obese people. Available antiobesity pharmaceuticals are only a few, and their efficacy is not good or has unavoidable side effects that limit their use. Surgical interventions like bariatric surgery work well, but regain of weight after surgery cannot be prevented. This made ideal antiobesity natural medications that can be used safely on a regular basis to control weight gain and resulting comorbidities a necessity [4]. As herbal formulations were used in different traditional systems of medicine from prehistoric times as powerful healing approaches, recently, attention is being increased in those formulas to search for a safe and effective therapy [5, 6]. A combination of herbal medicines to form polyherbal formulations can be the best method to achieve a synergic effect as well as reduce the toxicity associated with a single phytochemical from one plant. Indeed, the use of herbal resources will be cost effective, ecofriendly, and readily available as well [6, 7]. The diversity of compounds in the polyherbal formula may act on several targets at the same time to get rid of diseases. Due to this reason, the desired therapeutic outcomes can be obtained even at a lower dose of individual chemical components. Indeed, polyherbal formulations also improve patient compliance and therapeutic effect by limiting the need to intake multiple single herbal formulas at a time. Hence, polyherbal formulations are believed to give holistic treatment against multiple diseases [6, 8, 9]. In recent times, various scientific reports support the effectiveness of polyherbal formulations in controlling obesity and adipogenesis [10–14]. The herbal medicine showed antiobesity activity through various mechanisms such as inhibition of adipogenesis, suppression of food intake, increased energy expenditure by thermogenesis, inhibition of lipase activity, stimulation of diuresis, regulation of lipid metabolism, increased satiety, stimulation of insulin secretion, and action on the central nervous system through leptin [7, 15–17]. These herbal medicines are also added to nutraceutical sometimes and are regulated as food for special dietary use and food for special medicinal or therapeutic purpose for the treatment of subjects with overweight, obesity, and blood pressure, etc. [18].

In this research, we developed formulations A and B and evaluated the antiobesity activity. The formulation A was prepared by mixing *Citrus reticulata* Blanco [Rutaceae; fruit peel], *Cassia obtusifolia* L. [Fabaceae; seeds], *Houttuynia cordata* Thunb. [Saururaceae; aerial parts], *Benincasa hispida* (Thunb.) Cogn. [Cucurbitaceae; fruit peels], and *Aconitum ciliare* DC. [Ranunculaceae; leaves] along with root powder of *Helianthus tuberosus* L. [Asteraceae] (also known as synanthrin or inulin), whereas the formulation B was prepared by mixing *Acanthopanax gracilistylus* W.W.Sm. [Araliaceae; leaves], *Dioscorea oppositifolia* L. [Dioscoreaceae; root tubers], *Glycine max* (L.) Merr. [Fabaceae; seeds], *Phaseolus umbellatus* Britton [Fabaceae; seeds], *Momordica charantia* L. [Cucurbitaceae; fruits], and *Trichosanthes kirilowii* Maxim. [Cucurbitaceae; roots] along with the powder of *H. tuberosus* root. The antiobesity potential of *C. reticulata* [19], *C. obtusifolia* [20], *H. cordata* [21], *B. hispida* [22], *H. tuberosus* [23], *D. oppositifolia* [24], *G. max* [25], *M. charantia* [26], and *T. kirilowii* [27] has been separately and individually studied in the previous studies. However, the combined effect as a form of formulation of these medicinal herbs has not been evaluated. The main objective of this study was to investigate the antiobesity activity of formulations A and B in HFD-induced obese C57BL/6J mice.

## 2. Materials and Methods

### 2.1. Preparation and Extraction of Formulations A and B

*Formulation A*: *C. reticulata* (fruit peels; 30 g), *C. obtusifolia* (seeds; 9 g), *H. cordata* (aerial parts; 15 g), *B. hispida* (fruit peels, 20 g), and *A. ciliare* (leaves; 10 g) were mixed and pulverized into coarse powder of 40 mesh size. The obtained powder was refluxed in distilled water two times for 2 hr and filtered. Filtrates obtained were mixed and concentrated in a vacuum pump rotatory evaporator to get viscous residue, which on freeze-drying yielded brown powder. The obtained brown powder was then thoroughly mixed with 15 g of *H. tuberosus* root power (also known as synanthrin or inulin) to obtain formulation A.

*Formulation B*: The similar procedure as formulation A was followed to prepare formulation B. It was obtained by mixing *A. gracilistylus* (leaves; 15 g), *D. opposite* (root tuber; 20 g), *G. max* (seeds; 25 g), *P. umbellate* (seeds; 25 g), *M. charantia* (fruits; 25 g), and *T. kirilowii* (root; 10 g) along with 15 g of *H. tuberosus* root power.

### 2.2. *In Vitro* Antioxidant Assay

The methods explained by Choi et al. were followed to evaluate the DPPH (1,1-diphenyl-2-picrylhydrazyl) and ABTS (2,2′-azino-bis (3-ethylbenzothiazoline-6-sulfonic acid)) free radical scavenging potential of the formulations [28]. Antioxidant power was represented as the amount needed to reduce free radicals by 50% concentration (IC_50_), obtained by a logarithmic regression curve between free radical scavenging activity and the concentration of formulation.

### 2.3. *In Vivo* Antiobesity Activity

#### 2.3.1. Animal Grouping, Treatment Schedule, Food Intake, and Food Efficiency Ratio (FER)

Male C57BL/6J mice (all seven weeks old) were brought from the Central Lab Animal Inc., Seoul, Korea. They were then acclimatized for 1 week in a pathogen-free controlled lab environment with 24 ± 1°C room temperature, 50-60% relative humidity, 12/12 dark-light cycle, and tap water supply *ad libitum*. The animal experiment was approved by the Wonkwang University Animal Ethics Committee (approval number: WKU19-77). Mice were then divided into test groups randomly, after measuring their weight before the start of the experiment, each group containing five (*n* = 5) mice. The composition of the experimental diets used in this study is presented in Supplementary Table S1. The normal control group was fed with a standard chow diet (standard diet: 5L79; contained 13.67% fat, 21.03% protein, and 62.70% carbohydrate) from Orient Bio Inc., Seongnam, Korea. All other test groups were fed with a high-fat diet (HFD: Rodent Diet D12492, Research Diets, New Brunswick, NJ, USA), composed of 60% fat, 26.20% protein, and 26.30% carbohydrate. The normal group and HFD-fed control groups were given phosphate-buffered saline (PBS) as a blank twice a day (BID) orally using an oral feeding tube, while the test groups were fed with 100 mg/kg BID formulation A and 500 mg/kg BID formulation B using an oral feeding tube for a 12-week treatment period. PBS was used as carrier for suspending the prepared formula. Body weight and food intake were monitored every week throughout the experiment period. Food intake was calculated by deducting the remaining diet in each group cage by the total amount supplied to them. After completion of the experiment, the food efficiency ratio (FER) was calculated as follows:

(1) $$\begin{array}{c} \text{FER}\%=\left(\frac{\text{gained body weight }\left(\text{g}\right)}{\text{food intake }\left(\text{g}\right)\text{ during the experiment period}}\right)\times 100\%. \end{array}$$

At the end of the experimental period, the mice were fasted for 14 hours prior to sacrifice, and then, blood was collected under anesthesia. Collected blood was centrifuged at 14000 rpm for 20 minutes at 4°C to obtain the serum. The obtained serum was stored at -70°C to preserve for further biochemical analysis.

#### 2.3.2. Measurement of Mouse Blood Glucose Level

The blood glucose level was measured in 14 hr fasting conditions at the initial day of the experiment and thereafter in every 3-week interval by using a one-touch blood glucose monitoring system (CareSens® N, i-SENS, Korea) using the manufacturer protocol.

#### 2.3.3. Measurement of Serum Insulin

Mouse Insulin ELISA Kit (Cat#638-01489, Shibayagi Co., Ltd., Japan) was used to determine serum insulin following the manufacturer protocol. Fasting blood glucose on the sacrificed day and insulin level in the serum were used to calculate the homeostasis model assessment of insulin resistance (HOMA-IR) as follows:

(2) $$\begin{array}{c} \text{HOMA}‐\text{IR}=\frac{\text{fasting insulin}\left(\mu \text{U}/\text{mL}\right)\times \text{fasting glucose}\left(\text{mmol}/\text{L}\right)/}{22.5} \end{array}$$

#### 2.3.4. Measurement of Serum Lipid Profiles

Total cholesterol, high-density lipoprotein (HDL), low-density lipoprotein (LDL), and triglyceride amount were measured in the serum using a bioassay kit (BioVision, Milpitas, USA) following the provided manual. Atherogenic index (AI) can closely correlate with the cardiovascular risk of an obese subject [29]. AI was calculated using the formula

(3) $$\begin{array}{c} \text{Atherogenic Index}=\frac{\left(\text{Total Cholesterol}‐\text{HDL}\right)}{\text{HDL}} \end{array}$$

Percentage of cardio by formulation was calculated using the AI of the control and treatment group as follows:

(4) $$\begin{array}{c} \%\text{Protection}=\frac{\left(\text{AI of HFD control}‐\text{AI of treatment group}\right)}{\text{AI of control}*100} \end{array}$$

#### 2.3.5. Measurement of Lipid Content in Feces

Feces were collected from all treatment groups at the 2^nd^ week, 5^th^ week, and 10^th^ week after the start of treatment. For the collection of feces, mice were transferred to separate cage with bedding for a 2-hour period in the 14^th^, 35^th^, and 70^th^ days of the experiment, and feces defecated in the 2 hr duration in the new cage were collected and freeze dried. An equal amount of dry feces from every test group was taken and moisturized with 3 mL of water; homogenized and fat content was extracted with a mixture of methanol and chloroform (1 : 2) followed by quantification by measuring weight [30].

#### 2.3.6. Organ Weight and Histological Observations

After the sacrifice of experimental mice, the liver, epididymal white adipose tissue (WAT), kidney, and spleen were isolated to observe possible toxicity and extent of fat deposition in them. Isolated organs were immediately weighed and fixed into 10% neutral formalin solution for 2 days. Fixed tissues/organs were dehydrated in alcohol and embedded in paraffin. Thin tissue slices of tissue of about 5 *μ*m thicknesses were sectioned using a microtome, attached in a slide, and stained with hematoxylin and eosin (H and E). Stained slides were pictured using an EVOS XL core light microscope (Life Technologies, Bothell, WA, USA) at 10x and 20x magnifications.

### 2.4. Statistical Analysis

One-way analysis of variance (ANOVA) followed by Dunnett's multiple range test was used to determine statistically significant differences between the experimental groups using GraphPad Prism 7 software. The biological effects of formulations A and B were separately analyzed against the blank-treated control. Data are presented as the mean ± standard deviation (SD). Confidence interval above 95% (*P* < 0.05) was considered a statistically significant difference between groups.

## 3. Results

### 3.1. Antioxidant Activity of Formulations A and B

Antioxidant potentials of formulations A and B as represented by their ability to reduce free radical concentration (IC_50_) values are presented in Table 1. Formulation B was found to have good antioxidant potential than formulation A, but both of them have mild antioxidant potential than standard gallic acid.

The IC_50_ values were determined by using a logarithmic regression curve between free radical scavenging and the concentration of formulation.

### 3.2. Effect of Formulations A and B on Regulation of Mouse Body Weight, Food Intake, and Food Efficiency Ratio

**Figure 1:**
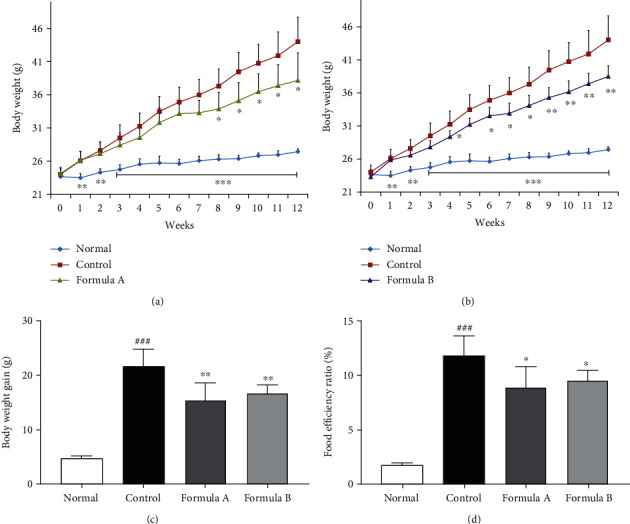
Effect of formulations A and B on body weight gains and food efficiency ratio in mice. Mouse body weights were measured every week over the experimental period. (a) Body weight pattern of formulation A. (b) Body weight pattern of formulation B. (c) The cumulative weight gain and (d) food efficiency ratio during the experimental period. The food efficiency ratio (FER) was calculated as follows: FER% = gained body weight (g) × 100/food intake (g). The data were shown relative mean expression ± SD. Statistical significance was calculated using one-way ANOVA followed by Dunnett's multiple comparisons test. ^∗^*P* < 0.05, ^∗∗^*P* < 0.01, and ^∗∗∗^*P* < 0.001 vs. control; ^###^*P* < 0.001 vs. normal.

Changes in the body weight patterns of mice in different groups throughout the 12 weeks of the treatment period are depicted in Figures 1(a) and 1(b). Change in body morphology after the treatment period can be observed in Supplementary Figure 1. The body weight in the HFD control group mice was drastically increased during the experimental period, which was significantly reduced by formulation treatment. The dramatically increased body weight gain at the end of the experiment due to HFD feeding was also reduced significantly by both formulations (Figure 1(c)). The food consumption pattern among HFD-fed groups was observed to be similar (Supplementary Figure 2). As a drastic increase in weight gain, the assessment of the food efficiency ratio (FER) also showed the significantly increased FER value in the control group compared to the normal group. However, the treated formulations significantly reduced the FER in comparison to the HFD control (Figure 1(d)). Inhibition of absorption of lipids from the gastrointestinal tract by local enzymatic inhibition as seen in orlistat is considered an important pathway for antiobesity agents [31]. Fat excretions in formulation A and B-treated groups were observed to be comparable to that in the control group. However, at the 10^th^ week of the treatment period, a slightly higher amount of fat was excreted in the drug treatment groups (Supplementary Figure S3). That signified that the formulations might have altered the gastrointestinal environment in a long-term application, inhibiting the fat absorption from the intestine.

### 3.3. Effect of Formulations A and B on Fasting Blood Glucose Level

**Figure 2:**
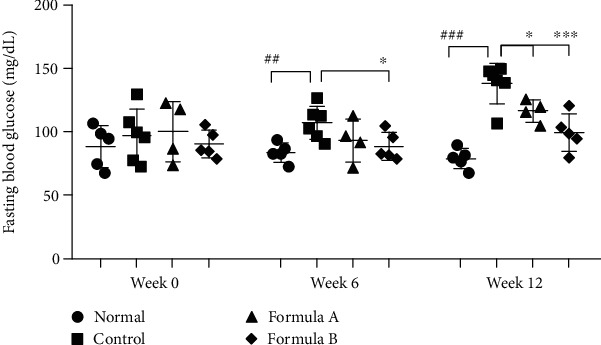
Effect of formulations A and B on fasting blood glucose level of mice. The data were shown relative mean expression ± SD. Statistical significance was calculated using one-way ANOVA followed by Dunnett's multiple comparison test. ^∗^*P* < 0.05 and ^∗∗∗^*P* < 0.001 vs. HFD control; ^##^*P* < 0.01 and ^###^*P* < 0.001 vs. normal.

Fasting blood glucose levels were measured at 3-week intervals, and the readings are presented in Supplementary Table S2. There was no significant difference in blood glucose level before the commencement of the experiment. As the experiment continues, significant difference between the groups was noticed. Blood glucose level increased significantly in the high-fat diet-fed group after 6 weeks of treatment. This increased glucose level was reduced significantly by both formulations as depicted in Figure 2.

### 3.4. Effect of Formulations A and B on Serum Insulin and HOMA-IR

**Figure 3:**
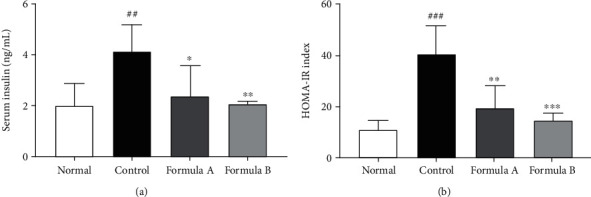
Effect of formulations A and B on serum insulin level and HOMA-IR. HOMA-IR index was calculated as follows: HOMA − IR = [fasting insulin (*μ*U/mL) × fasting glucose (mmol/L)]/22.5. The data were shown as relative mean expression ± SD. Statistical significance was calculated using one-way ANOVA followed by Dunnett's multiple comparison test. ^∗^*P* < 0.05, ^∗∗^*P* < 0.01, and ^∗∗∗^*P* < 0.001 vs. control; ^##^*P* < 0.01 and ^###^*P* < 0.001 vs. normal.

The serum level of insulin was measured in mice at the end of the experiment period. Insulin release and thus HOMA-IR were significantly increased in HFD control mice indicating the likelihoods of development of insulin resistance in those subjects due to HFD feeding. This increase in insulin and HOMA-IR was decreased by both formulations A and B significantly, indicating a possible preventive effect of the formulations on insulin resistance of treated mice (Figure 3).

### 3.5. Effect of Formulations A and B on Serum Lipid Profiles

**Figure 4:**
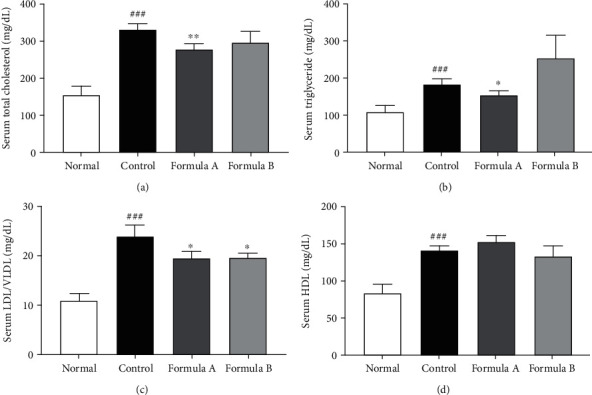
Serum lipid profile of total cholesterol, triglyceride, HDL, and LDL/VLDL on treatment with formulations A and B. The data were shown relative mean expression ± SD. Statistical significance was calculated using one-way ANOVA followed by Dunnett's multiple comparison test. ^∗^*P* < 0.05 and ^∗∗^*P* < 0.01 vs. control; ^###^*P* < 0.001 vs. normal.

Increases in serum levels of cholesterol, triglycerides, and LDL/VLDL (also known as bad cholesterol) were observed in the HFD control group in comparison to the normal group. This upsurge was reduced significantly by the treatment of formulation A as shown in Figure 4. However, formulation B only reduced LDL/VLDL significantly, and the alteration of other lipids was not statistically significant. Change in HDL (good cholesterol) for groups treated with both formulations was not statistically significant.

The significant increase in total cholesterol in the HFD control group caused an increase in the value of the atherogenic index (AI), which was observed to be lowered by formulation A giving about 39% protection. On the other hand, AI was only slightly reduced after treatment of formulation B, giving around 7% protection (Table 2).

Atherogenic index (AI) was calculated as AI = (total cholesterol − HDL)/HDL, and the percentage of protection was calculated as Protection (%) = (AI of HFD control − AI of treatment group)/AI of HFD control × 100.

### 3.6. Effect of Formulations A and B on Organ Weight and Histological Observations

**Figure 5:**
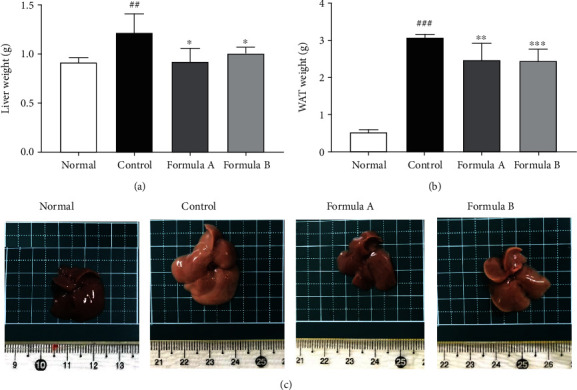
Effect of formulations A and B on visceral organs of mice. (a) Liver weight, (b) epididymal white adipose tissue weight, and (c) liver morphology of mice. The data shown were relative mean expression ± SD. Statistical significance was calculated using one-way ANOVA followed by Dunnett's multiple comparison test. ^∗^*P* < 0.05, ^∗∗^*P* < 0.01, and ^∗∗∗^*P* < 0.001 vs. control; ^##^*P* < 0.01 and ^###^*P* < 0.001 vs. normal.

**Figure 6:**
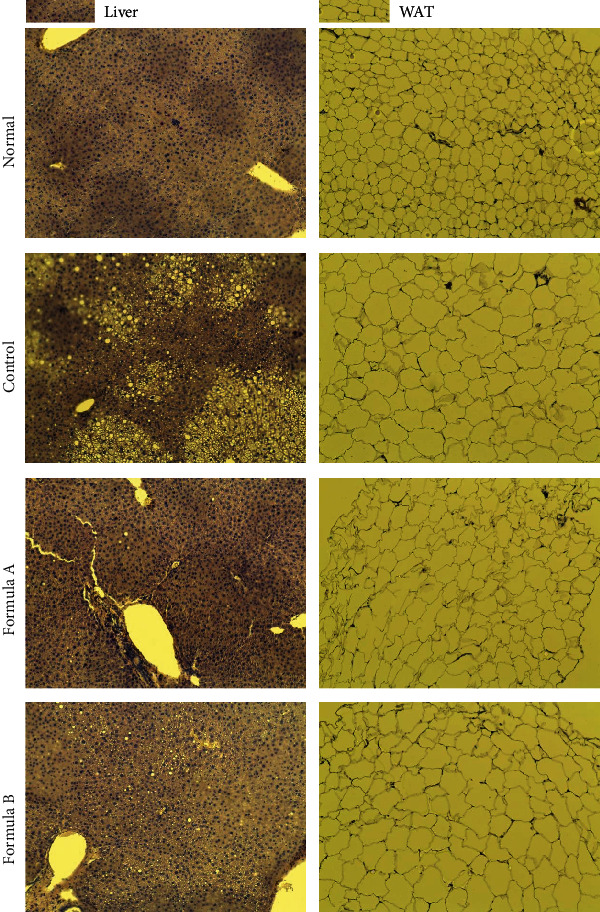
Histological observation of liver tissue and white adipose tissue after H and E staining. Micrographs were captured at 20x magnification under a light microscope. The liver of the normal diet feed group showed no sign of steatosis while high amounts of white fat droplets are visible in the HFD control group. Treatment of animals with formulations A and B prevents liver fat deposition. Similarly, WAT of normal diet feed animals has small compact adipose cells in them. In the control group, big and circular hypertrophic adipocytes were observed. Treatment with both formulations prevents hypertrophy of adipocytes keeping them in normal shape.

Liver and epidydimal white adipose tissue (WAT) weights were observed to be increased with 12-week feeding of HFD, which was significantly reduced by both formulations A and B (Figures 5(a) and 5(b)). The finding was further supported by histological observation of the liver revealing the absence of white fat droplets in the treated groups, indicating amelioration of hepatic steatosis (Figure 6). Histological observation of white adipose tissue also clearly indicated hypertrophy of adipocytes in the HFD-fed control group which was brought back to an almost normal level by both formulations (Figure 6). The effects of drug samples on the kidney and spleen were evaluated to observe the toxic response. Measurement of kidney weight showed that there was no significant difference between the drug-treated and control groups (Supplementary Figure 4A). Significant increases in the weight of the spleen were observed in the HFD-fed group (Supplementary Figure 4B). This might be because of the intracellular or intercellular fat deposition in the spleen due to HFD feeding [32]. Morphological and histologic observation of the liver, kidney, and spleen of the formulation-treated group did not show any sign of toxicity, indicating that the formulations are safe in the treated dose (Supplementary Figures S5 and S6).

## 4. Discussions

Lifestyle and behavioral modification, pharmacotherapy, and surgical intervention are among the famous strategies to overcome obesity. Lifestyle and behavioral therapy including increased physical activity and a high-quality diet with limited calories provide only limited benefit in losing weight for a person who already gained heavy weight. Moreover, the impact of diet and exercise interventions to reduce obesity and related disease burden was questioned by several findings [33–36]. Besides this, pharmaceutical intermediations such as liraglutide, empagliflozin, beloranib, and rimonabant were identified as giving good acute weight loss and prevention of obesity-related commodities, but the use of them is limited due to unavoidable side effects and rapid regain of weight after the termination of therapy [36, 37]. Lastly, surgical interventions like bariatric surgery and gastric bypass can treat obesity in the short term, but remission after some time cannot be prevented [38–40].

We developed two polyherbal formulations A and B by addressing all those limitations related to long-term safety and cost-effectiveness by using multiple commonly used herbs in a small amount together with *Helianthus tuberosus* (inulin), which was found to have a significant benefit in weight reduction by reducing glucose, triglyceride, and cholesterol and balancing overall lipid profile [41]. *H. tuberosus* contains a high amount of fiber and fructans and a diverse range of polyphenols while having negligible amounts of chemical contaminants, natural toxicants, and heavy metals [42, 43]. All selected medicinal plant samples in the formulations are popular herbal medicinal agents with established bioactivity. We expected the combination of pharmacologically active and regularly consumed herbal plant mixtures to give good bioactivity in mice.

The high-fat diet-induced obesity model in mice is considered a reliable method to understand human obesity as it resembles obese human subjects. Moreover, it can be used to study invasive and terminal investigations which would be impossible to perform in humans [44]. We evaluated the antiobesity activity of formulations A and B in this mouse model by monitoring weight, food intake, lipid excretion, and several biochemical parameters related to obesity and diabetes in obese mice.

Misbalance in the production of free radicals and antioxidant potential of the body causes oxidative stress. Oxidative stress and inflammation are responsible for several comorbidities in obesity. Thus, proper management of oxidative stress in time will reduce those adverse events in the body [45]. Both of our formulations have mild antioxidant effects (Table 1) and can serve as a herbal exogenous source of antioxidants to protect from oxidative stress and resulting comorbidities. This finding is further supported by literature showing antioxidant activity and identification of antioxidant molecules in *Citrus reticulata* [46], *Houttuynia cordata* [47], *Cassia obtusifolia* [48], and *Benincasa hispida* [49] of formulation A and *Acanthopanax gracilistylus* [50], *Dioscorea opposite* [51], *Momordica charantia* [52], and *Trichosanthis kirilowii* [53] of formulation B.

The extent to which body weight is increased by consuming a specific amount of food is known as the food efficiency ratio (FER). FER and hence mouse body weight was significantly reduced by both formulations A and B (Figure 1) indicating that less food was available for body weight gain compared to the high-fat diet control group. Reduced weight of the liver, epidydimal WAT, few fat droplet in the liver, and small size of adipocyte in WAT of the formulation-treated group compared to the HFD-fed control group also support less fat deposition by consumption of these formulations (Figure 6). Lower FER in the formulation-treated group might also be due to the increase in lipid excretion. Interestingly, the seed of *C. obtusifolia* from formulation A was found to inhibit pancreatic amylase and lipase and bind to bile acids impeding lipid absorption from the intestine [48, 54]. Similarly, the *C. reticulata* peel extract also inhibited amylase and glucosidase in rat models [55]. The anorectic activity of *Benincasa hispida* was observed, indicating less hunger and food intake after eating this fruit [22]. *Glycine max* [56], *Dioscorea opposite* [57], *Momordica charantia* [58], and *Trichosanthis kirilowii* [59] used in formulation B had shown inhibition of pancreatic amylase and/or glucosidase inhibition in several studies hindering lipid absorption from the intestine. So we can assume that combination of all those ingredients is responsible for the reduced food efficiency ratio of the formulation.

Obesity is correlated with the increased prevalence of type II diabetes mellitus. Impairment of glucose tolerance resulting from insulin resistance is considered to be a preliminary indication for type II diabetes mellitus [60]. To examine insulin resistance and impairment in glucose metabolism, we evaluated the fasting blood glucose level and serum insulin level and calculated the HOMA-IR ratio in experimental animals. Significant decrease in the fasting blood glucose level (Figure 2), serum insulin (Figure 3(a)), and, hence, the HOMA-IR (Figure 3(b)) index showed that the formulations' treatments helped to balance disrupted glucose metabolism and possibly insulin resistance in diet-induced obese mice indicating their potential antidiabetic activity. Reduction of fat droplet in histological evaluation of the liver and reduction of size of adipocyte in WAT also support increased fat deposition in high-fat diet-fed subjects (Figure 6).

It was found that the lipid profile gets disrupted in obese subjects predisposing them towards cardiovascular diseases. Controlling lipid profile and obesity in time is believed to prevent an adult from life-threatening cardiovascular problems [61]. Our evaluation of the lipid profile showed that formulation A significantly decreased cholesterol, triglycerides, and LDL/VLDL while increasing HDL levels (Figure 4). Formulation A showed decreased atherogenic index while providing 38.7% cardioprotection (Table 2). Significant reduction of LDL/VLDL and the atherogenic index was also observed with formulation B treatment, with 7.12% cardioprotection.

Obesity is accompanied by alterations in histological appearance, mainly steatosis of the liver [62], increased white adipose tissue mass [63], etc. Observation of significantly increased liver weight and fatty liver in the HFD control group was ameliorated by treatment of both formulations A and B (Figures 5(a) and 5(c)). Histological evaluation using H and E staining also revealed improvement of liver steatosis by both formulation treatments, evidenced by fewer white fat droplets in the liver tissue as shown in Figure 6. Further, white adipose tissue mass was also significantly reduced by the formulations' treatments (Figure 5(b)), decreasing the hypertrophy of adipocytes as observed by the small size of white adipose cells in histological observation (Figure 6).

Besides this, medicinal plants used in the formulations are already proven for a diverse range of bioactivities. A component of formulation A, the peel extract of *Citrus reticulata*, showed good antiobesity activity [19] and hypoglycemic activity [55]. Peels also contained a diverse range of phenolic and flavonoid compounds that make the peel a valuable source of bioactive compounds and nutraceuticals [46]. The seed of *Cassia obtusifolia* is a rich source of proteins and fiber [64]. *Houttuynia cordata* was used as a traditional Chinese medicine for a long time [65] and has hypoglycemic activity [66]. *Benincasa hispida*, a fruit with high nutritional value, contains amino acids, sugars, polyphenols, vitamins, fiber, and minerals [22]. Components of formulation B, *Acanthopanax gracilistylus* leaves, were established to have antidiabetic activity and contain a diverse range of polyphenols in them [67]. Dioscorea spp. had been the source of food since the forest-dwelling primitive life and cover around one-tenth of the root tubers in the current global market. They are rich sources of nutrition due to the presence of diverse polysaccharides, fibers, vitamins, and minerals [68]. Chinese yam (Dioscorea opposita) is a famous component of traditional Chinese medicine as well as has been shown to have hypoglycemic effects [69]. *Glycine max* (black soybeans) and Phaseolus umbellate (red beans) are well known for high nutritional value and health benefits like antidiabetic, antiaging, and neuroprotective effects [70–72]. Momordica charantia, also known as bitter gourd, has well-known health benefits including antidiabetic, anti-inflammatory, antihypertensive, and hypoglycemic effects [73, 74]. Trichosanthis kirilowii was also found to have hypoglycemic and hypolipidemic activities [53].

Taking consideration of all results and used ingredients, we can assume that their combined efforts may account for a good antiobesity effect in the animal study. Furthermore, there was no toxicological observation on treated mice on physical and histological evaluations in the liver, spleen, and kidneys (Supplementary Figures 5 and 6). The samples used were commonly eaten food/herbal products so can be considered safe for consumption. Further evaluation of these formulations in clinical subjects is recommended to establish the activity in humans.

## 5. Conclusion

This study demonstrated the antiobesity activity of two polyherbal formulations A and B in diet-induced obesity (DIO) models of mice. These formulations showed potential antiobesity effects by lowering the body weight of mice, mainly by reducing food efficiency ratios and blocking fat deposition in WAT and visceral organs. Treatment with formulations A and B ameliorates lipid misbalance and probably insulin resistance, giving excellent cardioprotection to mouse models. Therefore, these formulations can be considered as a safe and effective potential intervention for the management of obesity.

## Figures and Tables

**Table 1:** IC_50_ value of formulations A and B and gallic acid to scavenge DPPH and ABTS free radicals.

Samples	IC_50_ (*μ*g/mL) for DPPH assay	IC_50_ (*μ*g/mL) ABTS assay
Formulation A	56.86	27.52
Formulation B	50.04	20.62
Gallic acid	0.85	0.54

**Table 2:** Atherogenic index and percentage protection provided by formulations A and B.

Group	Atherogenic index (AI)	Cardioprotection (%)
Normal	0.92 ± 0.50	—
Control	1.37 ± 0.21	—
Formula A	0.84 ± 0.17	38.71
Formula B	1.28 ± 0.46	7.12

## Data Availability

The datasets used and/or analyzed during the current study are available from the corresponding authors on reasonable request.

## Supplementary Materials

Supplementary Materials Figure S1: morphology of mice on treatment with formulations A and B. Figure S2: mouse food intake pattern after treatment with 2 formulations. Figure S3: effects of formulations A and B on lipid excretion in feces in different days of treatment periods. Figure S4: kidney and spleen weight after treatment with formulations A and B. Figure S5: histology of kidney after completion of treatment periods (H and E staining). Figure S6: histology of spleen after completion of treatment period (H and E staining). Table S1: composition of experimental diets. Table S2: effect of formulations A and B on fasting blood glucose of mice in 3-week interval.
