# Supplementary material for: Antiobesity Activity of Two Polyherbal Formulations in High-Fat Diet-Induced Obese C57BL/6J Mice
Source: Biomed Res Int. 2022 May 11;2022:9120259. doi: 10.1155/2022/9120259 (PMC9192239; doi:10.1155/2022/9120259)
Supplement: Supplementary Materials — Figure S1: morphology of mice on treatment with formulations A and B. Figure S2: mouse food intake pattern after treatment with 2 formulations. Figure S3: effects of formulations A and B on lipid excretion in feces in different days of treatment periods. Figure S4: kidney and spleen weight after treatment with formulations A and B. Figure S5: histology of kidney after completion of treatment periods (H and E staining). Figure S6: histology of spleen after completion of treatment period (H and E staining). Table S1: composition of experimental diets. Table S2: effect of formulations A and B on fasting blood glucose of mice in 3-week interval. [file 9120259.f1.docx]

Supplementary Material

Anti-obesity Activity of Two Polyherbal Formulations in High-fat Diet-Induced Obese C57BL/6J Mice

Prakash Raj Pandeya^1^, Gopal Lamichhane^1^, Ramakanta Lamichhane^1^, Jiao Luo ^2^, Xiao-Jun Li ^3^, Su-jin Rhee^4^, Liu Xiangqian^2^ and Hyun-Ju Jung^1^

1. Department of Oriental Pharmacy and Wonkwang-Oriental Medicines Research Institute, Wonkwang University, Sinyong-Dong, Iksan, 570-749, South Korea; [pandeya.praj@gmail.com](mailto:pandeya.praj@gmail.com) (P. R. P.), [lamichhanegopal1@gmail.com](mailto:lamichhanegopal1@gmail.com) (G.L.), [clickrama@hotmail.com](mailto:clickrama@hotmail.com) (R. L.)
2. School of Pharmacy, Hunan University of Chinese Medicine, Changsha 410208, China; [744561218@qq.com](mailto:744561218@qq.com) (J. L.)
3. National Engineering Research Center for Modernization of Traditional Chinese Medicine - Hakka Medical Resources Branch, School of Pharmacy, Gannan Medical University, Ganzhou, Jiangxi 341000, China; [xjli@gmu.edu.cn](mailto:xjli@gmu.edu.cn) (X-J. L)
4. Department of Pharmacy, Wonkwang University, Sinyong-Dong, Iksan, 570-749, South Korea; [rheesj05@wku.ac.kr](mailto:rheesj05@wku.ac.kr) (S-J. R.)

Correspondence should be addressed to Liu Xiangqian; [lxq0001cn@163.com](mailto:lxq0001cn@163.com) and Hyun-Ju Jung; [hyun104@wku.ac.kr](mailto:hyun104@wku.ac.kr)

Prakash Raj Pandeya and Gopal Lamichhane contributed equally to this work.

**Supplemental Figures**

**Figure S1**


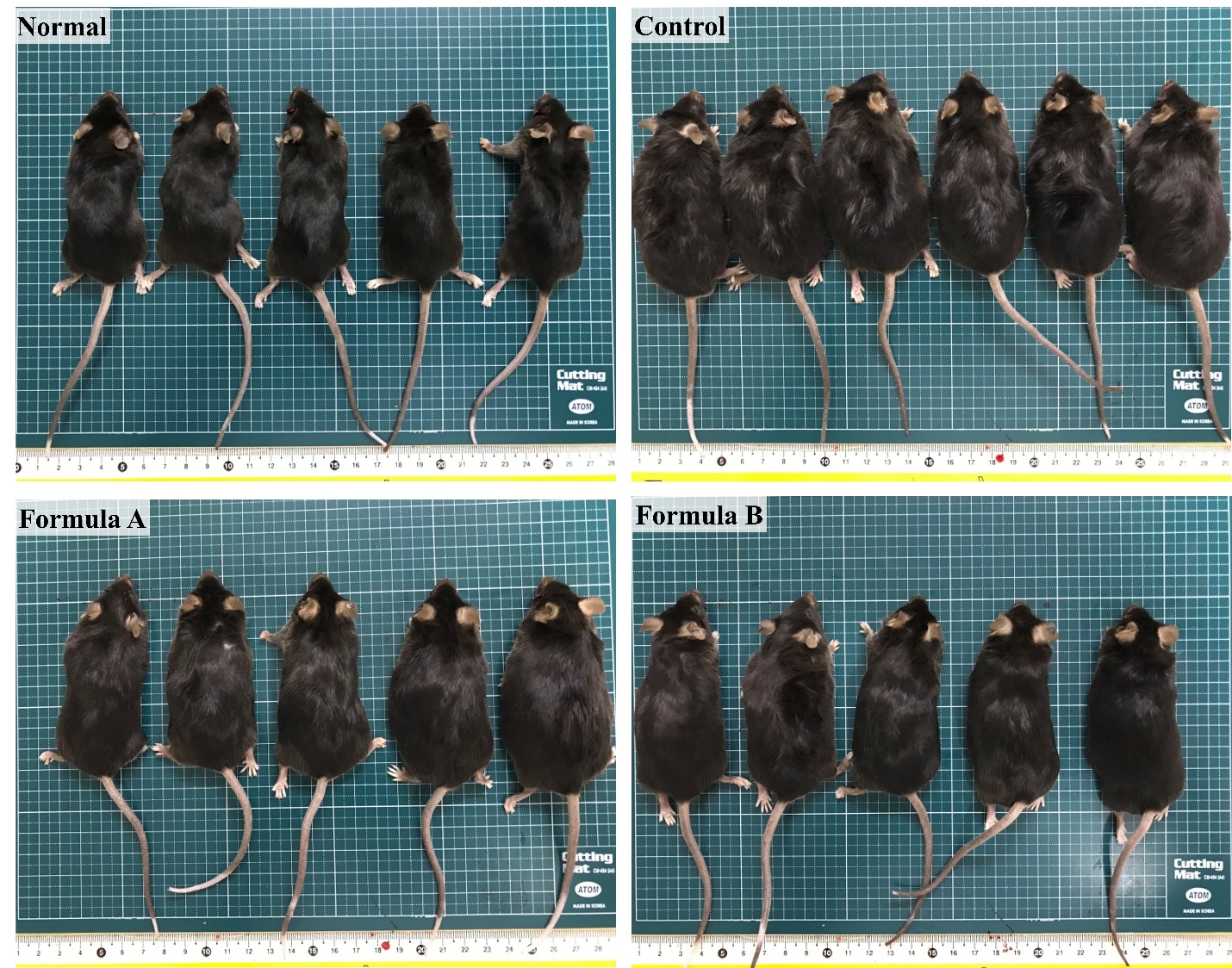


**Figure S1: Morphology of mice on treatment with formulation A and B.**

**Figure S2**


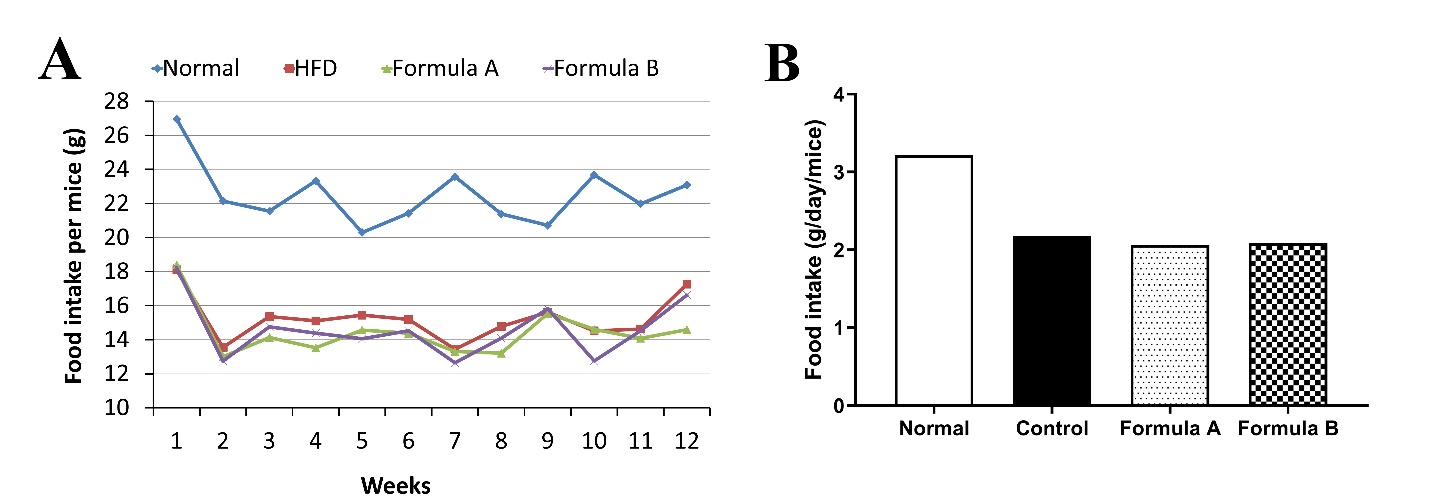


**Figure S2: Mice food intake pattern after treatment with 2 formulations.**

**Figure S3**


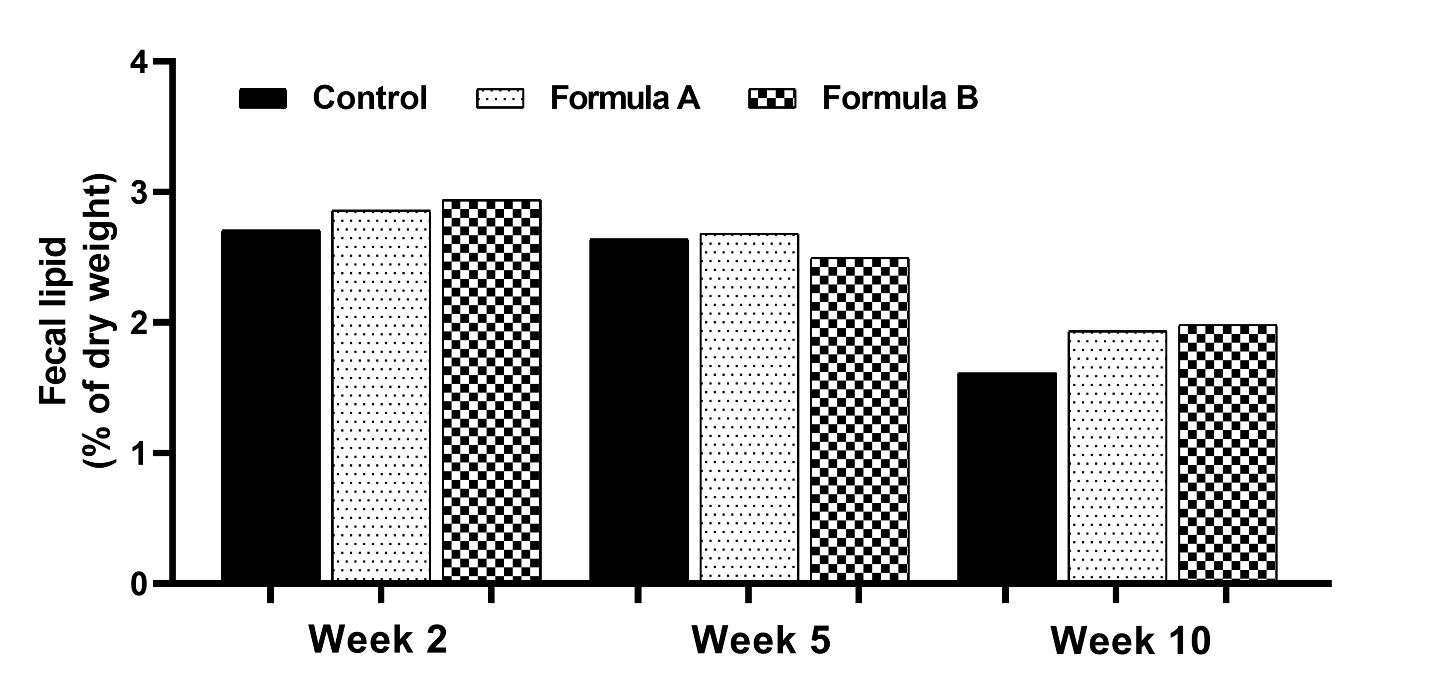


**Figure S3: Effects of Formulation A and B on Lipid Excretion of lipid in feces in different days of treatment periods.**

**Figure S4**


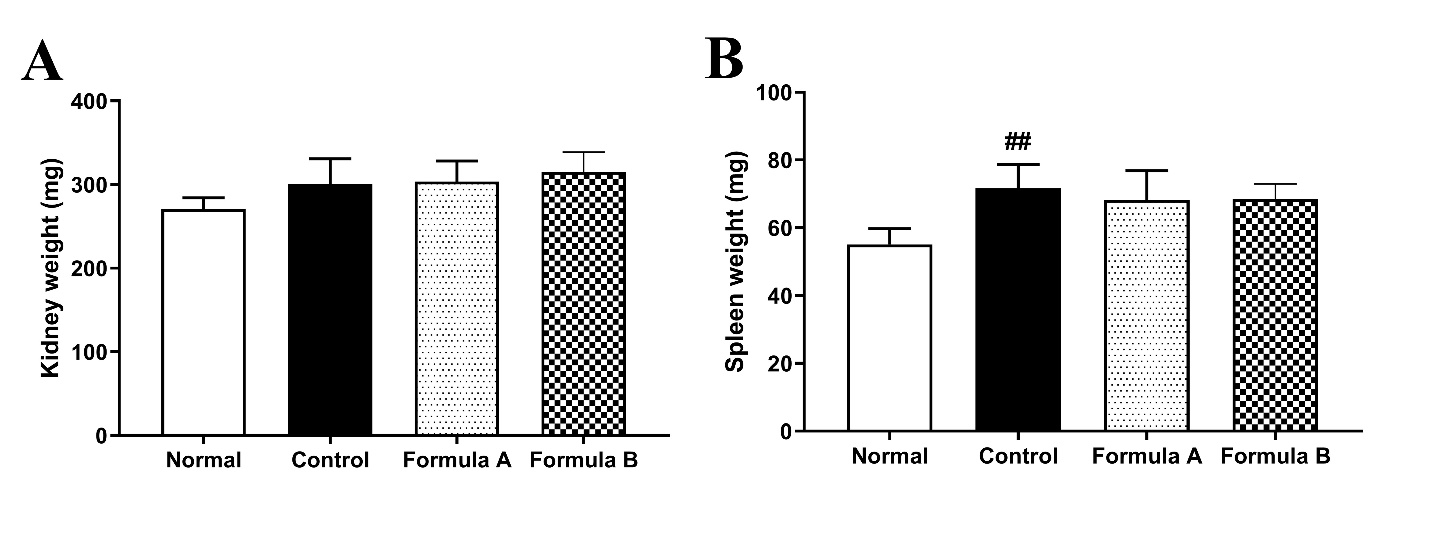


**Figure S4: Kidney and spleen weight after treatment with formulations A and B.**

**Figure S5**


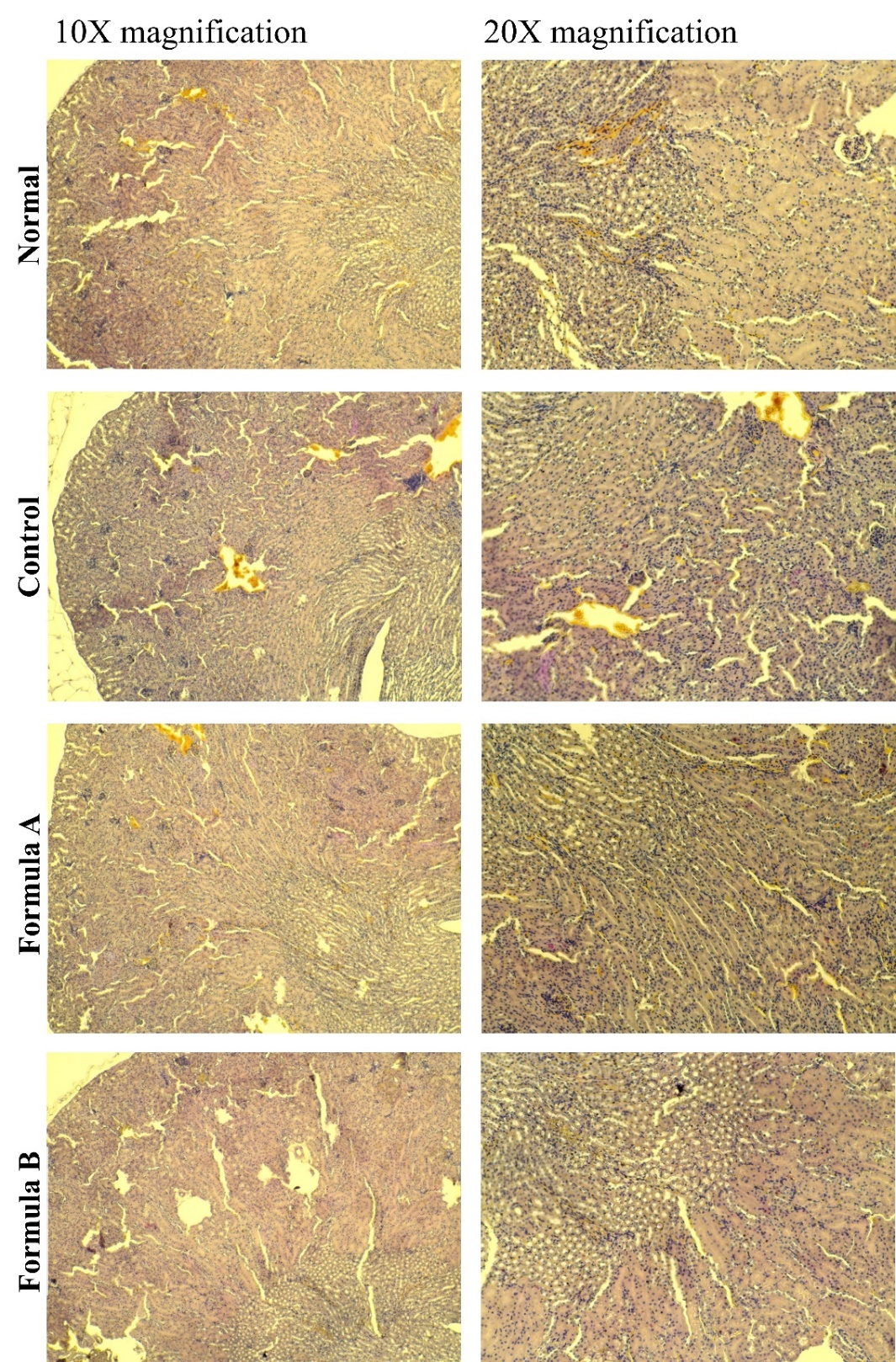


**Figure S5: Histology of kidney after completion of treatment periods (H and E staining).** Pictures were captured at 10X and 20X magnification under a light microscope. Glomerular and tubular lipid ambulation, expansion of Bowman’s space in capsule, and macrophage infiltration were evaluated. No significant changes were observed among experimental groups.

**Figure S6**


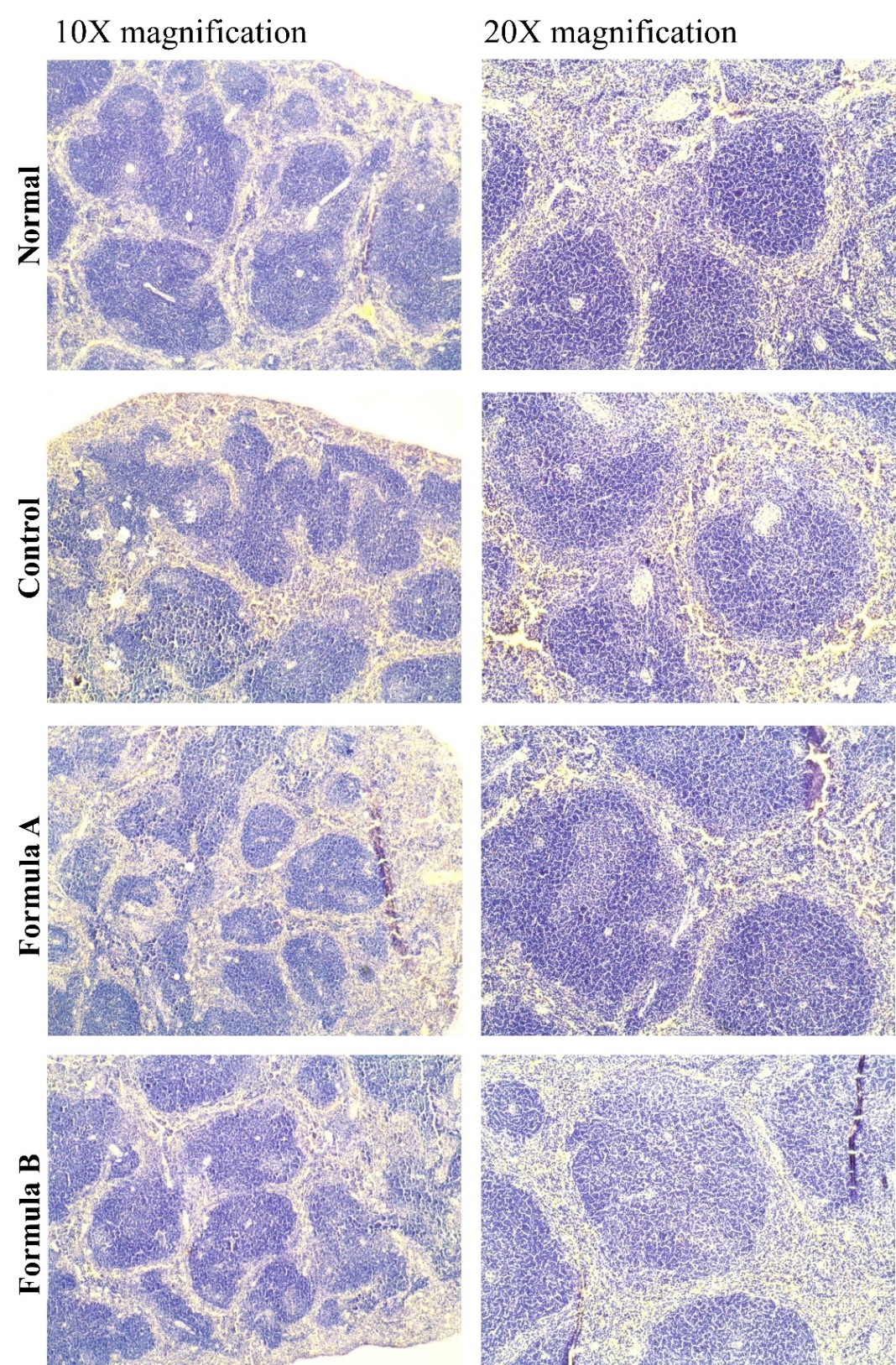


**Figure S6: Histology of spleen after completion of treatment period (H and E staining).** Pictures were captured at 10X and 20X magnification under light microscope. Histological architectures of white and red pulp and lipid accumulations were evaluated. No significant changes were observed among experimental groups.

**Supplemental Tables**

Table S1. Composition of experimental diets

| **Composition of standard chow diets** | | **Composition of high-fat diets** | |
| --- | --- | --- | --- |
| **Ingredients** | **Composition (%)** | **Ingredients** | **Composition (g/kg)** |
| Crude fiber | 6.7 | Casein | 200.0 |
| Amino acids | 18.4 | l-Cystine | 3.0 |
| Cholesterol (ppm) | 103 | Maltodextrin 10 | 125.0 |
| Total saturated fatty acids | 1.7 | Sucrose | 68.8.0 |
| Total monosaturated fatty acids | 1.4 | Lard | 245.0 |
| Starch | 33.9 | Soybean oil | 25.0 |
| Glucose | 0.3 | Cellulose BW200 | 50.0 |
| Fructose | 0.3 | Mineral mix S10026 | 10.0 |
| Sucrose | 2.0 | Calcium carbonate | 5.5 |
| Lactose | 0.3 | Potassium citrate | 16.5 |
| Calcium | 0.85 | DiCalcium Phosphate | 13.0 |
| Phosphorus | 0.62 | Vitamin mix V10001 | 10.0 |
| **Nutritional facts: % g (w/w) (% kcal)** | | | |
| **Protein** | 18.0 (20.1) | **Protein** | 26.2 (20.0) |
| **Carbohydrate** | 62.7 (65.30) | **Carbohydrate** | 26.3 (20.0) |
| **Fat** | 5.2 (13.67) | **Fat** | 34.9 (60.0) |

**Table S2: Effect of formulation A and B on fasting blood glucose of mice in 3 weeks interval**

| **Groups** | **Blood glucose level (mg/dL)** | | | | |
| --- | --- | --- | --- | --- | --- |
|  | **Week 0** | **Week 3** | **Week 6** | **Week 9** | **Week 12** |
| Normal | 88.80 ±16.56 | 92.40 ±9.15 | 84.00 ±7.62 | 71.80 ±6.26 | 79.40 ±7.99 |
| Control | 97.50 ±20.76 | 99.67 ±23.92 | 107.50 ±13.08^##^ | 114.00 ±14.09^###^ | 138.33 ±15.90^###^ |
| Formula A | 100.50 ±23.78 | 96.00 ±27.68 | 93.50 ±16.90 | 109.00 ±12.11 | 116.75 ±8.85* |
| Formula B | 90.80 ±10.94 | 85.00 ±13.95 | 89.00 ±11.07* | 94.60 ±9.81* | 99.80 ±14.86*** |
